# Infection on Frail Patients in the Intensive Care Unit: Insights From the PalMuSIC Study

**DOI:** 10.7759/cureus.63897

**Published:** 2024-07-05

**Authors:** Iuri Correia, Susana Fernandes, Mariana Bernardino, João Gonçalves Pereira

**Affiliations:** 1 Internal Medicine Department, Hospital Professor Doutor Fernando Fonseca, Amadora, PRT; 2 Palliative Medicine Unit, Hospital CUF Tejo, Lisbon, PRT; 3 Intensive Care Department, Hospital de Santa Maria, Lisbon, PRT; 4 Intensive Care University Clinic, Faculdade de Medicina da Universidade de Lisboa, Lisbon, PRT; 5 Intensive Care Unit Department, Hospital de Vila Franca de Xira, Vila Franca de Xira, PRT

**Keywords:** antibiotic therapy, multidrug resistance, intensive care unit, infection, clinical frailty

## Abstract

Background: Along with population aging, frailty is also increasingly common in the intensive care unit (ICU). However, the impact of frailty on the infection incidence, the risk of multidrug-resistant (MDR) microorganisms, and the potential benefits of broad-spectrum antibiotics are still poorly studied.

Methods: This is a multicentric, prospective, observational study collecting data for 15 consecutive days of all consecutive adult patients admitted in each participating ICU. Exclusion criteria included admission for less than 24 hours or failure to obtain informed consent. The Clinical Frailty Score (CFS) was calculated both by the doctor and by the nurse in charge, and the patient's next of kin. Patients were considered frail if the mean of the three measured scores was ≥5. This is a post hoc analysis of the PALliative MUlticenter Study in Intensive Care (PalMuSIC) study. The Hospital de Vila Franca de Xira Ethics Committee approved the study (approval number: 63).

Results: A total of 335 patients from 23 Portuguese ICUs were included. Frailty was diagnosed in 20.9%. More than 60% of the patients had a diagnosis of infection during their ICU stay, either present on admission or hospital-acquired. This included 25 (35.7%) frail and 75 (28.3%) non-frail (p=0.23) patients diagnosed with infection. In 34 patients, MDR microorganisms were isolated, which were more common in frail patients (odds ratio (OR): 2.65, 95% confidence interval (CI): 1.3-5.6, p=0.018). Carbapenems were started in 37 (18.1%) patients, but after adjusting for frailty and severity, no clear mortality benefit of this strategy was noted (odds ratio for ICU mortality: 1.61, 95% confidence interval: 0.49-5.31, p=0.43; odds ratio for hospital mortality: 1.61, 95% confidence interval: 0.61-4.21, p=0.33).

Conclusion: Frail patients had similar rates of infection to non-frail patients but were more prone to have MDR microorganisms as causative pathogens. The use of empirical therapy with large-spectrum antibiotics should be based on microbiological risk factors and not simply on the host characteristics.

## Introduction

Both pre-frailty and frailty are highly prevalent throughout Europe. It has been shown that almost 43% of the European population aged ≥50 years is in a state of pre-frailty and almost 8% is already frail [1]. In the intensive care unit (ICU), frailty is generally measured using the Clinical Frailty Score (CFS) [2], a 9-point scale developed to define adults' general level of frailty, with higher scores signifying greater frailty. Frailty has been associated with worsened outcomes in the ICU [3].

Frail individuals find it more difficult to return to their baseline state of health after a stressor [4]. Several physiological principles are shared by aging and frailty, leading to the loss of proper coping with challenges to homeostasis [5]. Sepsis is among the most common stress events in old patients and increases significantly with age, not only in frequency but also in associated lethality [5].

Nevertheless, several clinical situations may mimic sepsis, especially in old and frail patients, with a tendency for widespread antibiotic use [6], directly related to the gradual increase in resistant microorganisms [7]. The use of antibiotics significantly affects the patient's microbiota, causing dysbiosis and facilitating the selection of resistant microorganisms. This has a significant negative impact on the patient's health and hospital ecology.

In the ICU, the prescription of broad-spectrum antibiotics as empirical therapy is common. This seems to be mostly due to illness severity and the high rates of multidrug-resistant (MDR) pathogen colonization or infection [8]. The antibiotics used to treat MDR infections are limited, expensive, potentially more toxic, and often require hospitalization for parenteral administration. All these are particularly harmful to the frail patient population. Besides, ICU patients frequently experience antibiotic-associated adverse medication events, mainly because of the underlying severe illness that increases their vulnerability to organ damage [9]. Moreover, the patient's immunosenescence, immunosuppression, and frailty seem to favor these microorganisms' persistence of infection or colonization [10].

In this study, we address the impact of frailty on infection incidence, the risk of MDR microorganisms, and the potential benefits of broad-spectrum antibiotics in a population of critically ill patients.

## Materials and methods

This is a pre-planned sub-analysis of the PALliative MUlticenter Study in Intensive Care (PalMuSIC) study. The study protocol is described elsewhere [11]. Briefly, PALliative MUlticenter Study in Intensive Care (PalMuSIC) is a prospective, observational, multicenter study. The Ethics Committee of the Hospital de Vila Franca de Xira approved the study (approval number: 63). The study aimed to evaluate the use of both palliative care consultation and invasive interventions in frail patients admitted to intensive care units in Portugal.

The 335 consecutive adult patients admitted for more than 24 hours to each of the participating 23 centers, during a 15-day consecutive period between March and May 2019, were included. Written informed consent was obtained from all patients or their representatives.

Patients' general demographic characteristics were evaluated. The ICU and hospital length of stay (LOS) were calculated, along with the need for organ support, the presence of do not resuscitate (DNR), or the limitation of therapeutic efforts orders. Patients' comorbidities and admissions to the hospital in the past three months were assessed. Mortality was evaluated at ICU discharge, hospital discharge, and after six months follow-up.

Frailty was assessed on admission to the ICU, using the CFS, independently by the nurse and doctor in charge, as well as by the most significant family member. According to the average of the three assessments, all patients were classified as frail (CFS≥5) or non-frail (CFS<5).

All included patients were screened for infection or antibiotic therapy during the ICU stay. Infection was diagnosed by the clinician in charge of the patient. All microbiological documentation and the presence of multidrug-resistant (MDR) microorganisms were reviewed. The selected antibiotic was registered. Antibiotics were divided into "carbapenems," "other beta-lactams" (such as penicillin and cephalosporins), and "others" (e.g., vancomycin and macrolides). We classified as "carbapenem" all patients who received these drugs, even when they also received other antibiotics. All patients who received a beta-lactam other than a carbapenem were classified as "beta-lactam." All other patients who received at least one other antibiotic were classified as "other."

The cause of admission to the ICU was also analyzed to check if the patients had sepsis criteria on admission.

Statistics

Data was summarized as mean ± standard deviation or median (25%-75% interquartile range (IQR)), according to data distribution. Categorical variables were described as numbers (%). The chi-square test was used to compare categorical variables, while continuous variables were evaluated using the Student's t-test or Mann-Whitney U test, according to data distribution. Odds ratios (ORs) with 95% confidence intervals (CIs) were computed. We developed a logistic regression analysis to assess the association between the prescription of carbapenems in ICU and hospital mortality, adjusting for severity, presence of multidrug-resistant microorganisms, and frailty. Odds ratios (ORs) with 95% confidence intervals (CIs) were computed. Model fit was assessed using the Hosmer-Lemeshow goodness-of-fit test.

Statistical analysis was performed using SPSS version 29.0 (IBM SPSS Statistics, Armonk, NY). All statistics were two-tailed, and the significance level was defined as p<0.05.

## Results

Demographics

A total of 368 patients were assessed, and 335 (66% male) were included in the final analysis. Of the 33 patients excluded, 29 were hospitalized for <24 hours in the ICU and four refused informed consent.

We found 70 (20.9%) frail patients (CFS≥5). Overall, the whole population's mean frailty score was 3.5±1.7. The general characteristics of the population, according to the presence of frailty, are shown in Table 1.

**Table 1:** Patients' general demographics according to frailty Data presented as number (%) or mean + standard deviation or median (interquartile range) according to data distribution. *Chi-square test unless otherwise stated, **Mann-Whitney U Test, #Student's t-test, ‡patients discharged alive from the hospital, only ICU: intensive care unit, LOS: length of stay, IMV: invasive mechanical ventilation, RRT: renal replacement therapy, MDR: multidrug resistant, DNR: do not resuscitate, LTE: limitation of therapeutic efforts order

	Frail	Non-frail	P*
All patients	70 (20.8%)	265 (79.2%)	
Age	71.4±12.9	61.0±17.0	<0.001^#^
ICU LOS	6 (9)	5 (7)	0.367^**^
Hospital LOS (ward)	18.5 (30.25)	13 (22)	0.045^**^
Hospital admission (previous 3 months)	25 (35.7%)	50 (18.9%)	0.004
DNR order	17 (24.3%)	21 (7.9%)	<0.001
LTE	18 (25.7%)	21 (7.9%)	<0.001
IMV	45 (64.3%)	170 (64.2%)	0.95
Non-IMV	13 (18.6%)	44 (16.6%)	0.72
RRT	17 (24.3%)	31 (11.7%)	0.012
Vasopressors	47 (67.1%)	139 (52.5%)	0.03
Infection	40 (57.1%)	162 (61.1%)	0.585
Sepsis	0 (0%)	20 (7.5%)	0.044
Septic shock	25 (35.7%)	55 (20.7%)	0.127
MDR microorganism	13 (18.6%)	21 (7.9%)	0.013
ICU mortality	11 (15.7%)	24 (9.1%)	0.123
Hospital mortality	27 (38.6%)	51 (19.2%)	0.001
6 months mortality	35 (50%)	63 (23.8%)	<0.001
Hospital readmission (first 6 months)^‡^	21 (48.8%)	53 (24.9%)	0.003

The length of stay in the ICU did not differ between frail and non-frail patients, but frail patients stayed in the ward (after ICU discharge) significantly longer than non-frail patients (18.5 (30.25) days versus 13 (22) days, Mann-Whitney U test, p=0.045) (Table 1).

Infection

As many as 202 (60.3%) patients were diagnosed with an infection during their ICU stay, with 100 (29.9%) patients being admitted for sepsis. Infection incidence and admission for sepsis did not differ between frail and non-frail patients (p=0.585 and p=0.228, respectively) (Table 1).

Microbiologically documented infections were reported in 116 patients; roughly one-third were Gram-positive. Of these, there were 34 (29.3%, 8 Gram-positive) with an isolated MDR microorganism. These were more frequently isolated from frail patients (18.6% versus 7.9%, p=0.013). Frailty was an independent risk factor for MDR microorganisms (OR: 2.65, 95% CI: 1.3-5.6, p=0.013). At least one hospital admission in the previous three months was reported in 35.7% of frail patients and 18.8% of non-frail patients, but these were not significantly associated with the presence of MDR microorganisms (p=0.056).

A total of 23 patients out of the 38 (60.5%) DNRs received at least one antibiotic (Table 2) during their ICU stay.

**Table 2:** Antibiotic prescription according to patients' characteristics Data presented as numbers (% within the condition). DNR: do not resuscitate, MDR: multidrug resistant

	Number	Carbapenems	Other beta-lactam	Other
All patients	204	37 (18.1%)	145 (71.1%)	22 (10.8%)
Frail patients	41	11 (26.8%)	26 (63.4%)	4 (9.8%)
Non-frail patients	163	26 (16%)	119 (73%)	18 (11%)
MDR bacteria	32	17 (53.1%)	10 (31.3%)	5 (15.6%)
DNR patients	23	5 (21.7%)	18 (78.3%)	0 (0%)

Carbapenems were selected in 18.1% of patients, including 26.8% of frail patients. Not surprisingly, more than half of the patients with isolated MDR bacteria received a carbapenem. Monotherapy was selected in 64.4% of patients, including 54.1% of those who received a carbapenem.

According to our multivariate model (Table 3), after adjusting for Simplified Acute Physiology Score (SAPS) II, the presence of frailty and MDR microorganisms, using a carbapenem was not associated with a decrease in either ICU or hospital mortality (Table 3). The ICU LOS was also similar (carbapenems 10 (12.5) versus non-carbapenems 7 (8), Mann-Whitney U test, p=0.052).

**Table 3:** Multivariate analysis for ICU mortality (A) and hospital mortality (B) Only patients with a diagnosis of infection were included. Hosmer-Lemeshow test: p=0.83 (model A) and p=0.72 (panel B) ICU: intensive care unit, SAPS: Simplified Acute Physiology Score, OR: odds ratio, CI: confidence interval

Panel A (ICU mortality)			
	OR	95% CI	P
SAPS II	1.075	1.04-1.11	<0.001
Multidrug resistant	0.854	0.24-3.04	0.808
Frailty score	0.956	0.72-1.27	0.758
Carbapenem	1.613	0.49-5.31	0.432
Panel B (hospital mortality)			
	OR	95% CI	P
SAPS II	1.062	1.04-1.09	<0.001
Multidrug resistant	0.616	0.22-1.76	0.365
Frailty score	1.190	0.94-1.50	0.142
Carbapenem	1.607	0.61-4.21	0.334

Outcome

As presented in Table 1, 15.7% of frail and 9.1% of non-frail patients died in the ICU, and 38.6% of frail patients and 19.2% of non-frail patients died in the hospital. When addressing only infected patients, ICU mortality was not significantly different between frail and non-frail patients (20.0% versus 11.1%, p=0.184), but both hospital mortality (40.0% versus 22.2%, p=0.027) and six-month mortality (50.0% versus 28.8%, p=0.013) were significantly higher in frail patients.

The infected and non-infected patients commonly needed hospital readmission within the first six months after hospital discharge (24.8% versus 24.7%); readmissions were more common in the frail population (48.8% versus 24.9%, p=0.03). Discharge home was possible in 70.9% of the non-frail patients and only 44.3% of the frail population.

## Discussion

In this study, we found that 60.9% of patients admitted to the ICU had an infection, either on admission or during their ICU stay. Furthermore, 29.9% of patients were admitted for sepsis. However, we did not find any difference in infection rate related to frailty. The prevalence of MDR microorganisms was higher in frail patients; this population has been hospitalized more often in the previous three months. Carbapenems were used as first-line therapy in 18.1% of patients. We did not find a clear benefit of this strategy, either in the frail or non-frail population.

The critically ill population often receives at least one antibiotic during their ICU stay [9], a finding which was also noted in our study. The decision to start an antibiotic can be a difficult one, especially for frail patients. The dangers of over- or undertreating must be carefully considered. Frail patients may exhibit unusual signs of infection, which might complicate the diagnosis and the prognosis of an infection and call for a high level of suspicion [12]. Physicians often tend to err on the side of caution, overtreating patients. Nevertheless, this is also not without risks [13]. Frail patient care commonly requires a complex strategy. Recovery is often slower and sometimes incomplete. The fear of infection in this population repeatedly leads to the overuse of antibiotics, especially large spectrum, that may not have the expected benefits. In our population, frail patients had a similar prevalence of infection and sepsis to non-frail patients, although they were more prone to have isolated MDR microorganisms. Curiously, although frail patients more often have previous hospital admissions, this risk factor was not independently associated with MDR microorganisms in our population. This might be associated with the common use of antibiotics in the ambulatory setting, also prone to promoting the development of MDR microorganisms [13] and that may have faded the impact of previous hospital admissions.

Infected frail patients had similar ICU mortality to non-frail but higher hospital mortality and six-month mortality, a pattern very similar to that of the whole population. Similar findings were observed in a recent Japanese study: a divergent evolution of mortality [14], mostly related to frailty and not so much to infection, itself. Besides, patients admitted to the ICU with sepsis remain at a greater risk of dying after being discharged [15], and this may impact mostly the frail and the older population [5].

The use of a large-spectrum antibiotic for every infection at the frontline can be misguided, and some studies have shown an excess of inappropriate antibiotic therapy with the common use of these very large-spectrum antibiotics in the ICU [9,16]. A vicious cycle of resistance and overuse of large-spectrum antibiotics is easily envisaged, leading to an increase in difficult-to-treat bacteria and new resistance. The overuse of the recently introduced antibiotics with an even larger spectrum may be doomed to fail to bring clear benefits if good stewardship of antibiotics is not set in place at the same time [17].

A palliative care strategy in the ICU setting, especially for frail patients, may help approach the therapeutic efforts according to the patient's goals of care and improve comfort measures rather than aggressive treatment [11,18,19]. This is particularly important for frail patients at the end of their lives, where the risks of these drugs may outweigh their potential benefits [10]. Although including antibiotics in therapeutic simplification is recommended, its practice is far from common [20].

Antibiotic stewardship in the ICU should include consideration for palliative care and avoiding futile antibiotics. This, along with the enhancement of infection prevention measures and surveillance for MDR microorganisms, may help mitigate the spread of antibiotic resistance in the ICU setting.

Limitations

This was a Portuguese multicenter study. No specific evaluation of patients for the presence of MDR microorganisms was performed, which may have influenced our results. Further, we did not collect data regarding the focus of infection. Besides, time on antibiotics, the decision to stop, and symptom resolution were not assessed.

The timing of the limitation of therapeutic efforts and DNR orders was also not registered, which limits the assumptions related to antibiotic use in those patients. Nevertheless, the fact that patients receiving a DNR order commonly received organ support during their ICU stay suggests that those orders more often were related to therapeutic failure than to patients' goals of care. Of the 38 patients who had a DNR order, 32 received invasive mechanical ventilation, 12 renal replacement therapy, and 27 vasopressors.

We also acknowledge that the inclusion of piperacillin-tazobactam in the "beta-lactam" group may have faded a potential benefit of the empirical use of carbapenems. We also did not collect data on the adequacy of empirical antibiotic therapy, and this limits our conclusions. However, a large recently published study has enlightened that using large-spectrum antibiotics when there is no clear microbiological risk for the presence of difficult-to-treat bacteria may lead to a worse outcome [21].

## Conclusions

Infection is frequent in frail patients in the ICU, but its prevalence is similar to that of the general population. Frail patients more often present MDR microorganisms and have high rates of previous hospital admissions, although these two were not related, possibly due to the common use of antibiotic therapy in the ambulatory setting. This population often receives large-spectrum antibiotics, although this may only provide an advantage if a microbiological risk is also present. Stewardship of antibiotics, along with a palliative care policy, should be in place to optimize patients' benefits.
